# More than a marker: potential pathogenic functions of MAP2

**DOI:** 10.3389/fnmol.2022.974890

**Published:** 2022-09-16

**Authors:** Rebecca A. DeGiosio, Melanie J. Grubisha, Matthew L. MacDonald, Brandon C. McKinney, Carlos J. Camacho, Robert A. Sweet

**Affiliations:** ^1^Department of Psychiatry, University of Pittsburgh, Pittsburgh, PA, United States; ^2^Department of Computational and Systems Biology, University of Pittsburgh, Pittsburgh, PA, United States; ^3^Department of Neurology, University of Pittsburgh, Pittsburgh, PA, United States

**Keywords:** MAP2, cytoskeleton, psychiatric disorder, neurodevelopment, neurodegeneration

## Abstract

Microtubule-associated protein 2 (MAP2) is the predominant cytoskeletal regulator within neuronal dendrites, abundant and specific enough to serve as a robust somatodendritic marker. It influences microtubule dynamics and microtubule/actin interactions to control neurite outgrowth and synaptic functions, similarly to the closely related MAP Tau. Though pathology of Tau has been well appreciated in the context of neurodegenerative disorders, the consequences of pathologically dysregulated MAP2 have been little explored, despite alterations in its immunoreactivity, expression, splicing and/or stability being observed in a variety of neurodegenerative and neuropsychiatric disorders including Huntington’s disease, prion disease, schizophrenia, autism, major depression and bipolar disorder. Here we review the understood structure and functions of MAP2, including in neurite outgrowth, synaptic plasticity, and regulation of protein folding/transport. We also describe known and potential mechanisms by which MAP2 can be regulated *via* post-translational modification. Then, we assess existing evidence of its dysregulation in various brain disorders, including from immunohistochemical and (phospho) proteomic data. We propose pathways by which MAP2 pathology could contribute to endophenotypes which characterize these disorders, giving rise to the concept of a “MAP2opathy”—a series of disorders characterized by alterations in MAP2 function.

## Introduction

The microtubule (MT) cytoskeleton is a fundamental coordinator of neuronal structure and function. MTs, comprised of heterodimers of α- and β-tubulin subunits, are present in all cell types and provide dynamic support to enable cell migration/division, modify and maintain cellular shape, and serve as tracks for intracellular trafficking (Goodson and Jonasson, 2018). In neurons, the MT network critically defines neurite morphology and mediates trafficking processes essential for synaptic transmission (Kapitein and Hoogenraad, 2015). This network is carefully regulated by a series of proteins which manipulate MT stability and/or arrangement.

Among these, the microtubule-associated proteins (MAPs) define a set of proteins which directly bind MTs, typically serving to stabilize them (Bodakuntla et al., 2019). MAPs additionally can have scaffolding functions, recruiting other cytoskeleton-modifying proteins or signaling pathway components to specific subcellular locations. Given the essential roles the MT cytoskeleton plays in neurons, it is unsurprising that pathology of both tubulin and various MAPs have been identified as direct precipitants of various brain disorders. The tubulinopathies, for instance, describe a set of cortical malformations caused by mutations in the several tubulin genes, while the tauopathies define a group of neurodegenerative diseases—most notably Alzheimer’s Disease—characterized by pathology of the axonal MAP Tau.

These conditions overlap in part with “opathies” generated by dysfunction of intrinsically disordered proteins (IDPs), which have several features that confer unique pathogenic potential (Box 1). The structurally fluid nature of IDPs, including Tau, makes them well suited to play roles in numerous, diverse signaling pathways (Dunker et al., 2005); thus, depending on the exact pathology, a range of pleiotropic effects can be observed in IDP pathogenesis. For instance, tauopathies are distinguishable by differences in the affected Tau isoform(s), affected cell type(s), and/or fibrillar Tau aggregate structure. A similar degree of heterogeneity exists in the synucleopathies, resulting from pathologies in the IDP alpha-synuclein. In such disorders, the IDP does not represent a specific locus of defined pathogenesis, but rather a shared hub of pathology which can lead to various effects depending on the exact nature of the pathology and what upstream factors precipitated it.

Box 1 **Features of intrinsically disordered proteins and their pathology.**

IDPs are proteins which lack a stable tertiary structure. They are present in all organisms, being particularly abundant in eukaryotes, where over 30% of all proteins are estimated to have disordered regions (Ward et al., 2004). Their ability to adopt a variety of transient conformations tailored to different interacting partners enables their engagement in both “one-to-many” and “many-to-one” signaling, as well as their ability to scaffold together proteins, e.g., components of a particular signaling pathway. IDPs are heavily modified by post-translational modification (Pejaver et al., 2014) and alternative splicing (Romero et al., 2006), and tend to have relatively low rates of synthesis and short half-lives (Gsponer et al., 2008), ensuring tight regulation of their expression and activity.

These features make IDPs efficient master regulators of diverse signaling pathways, but also leave them more prone to potential dysregulations which can give rise to disease. Genetic mutations or changes in expression, splicing, modifications, trafficking and/or degradation all can lead to aberrant complex formation, signaling activity, and/or folding. The “IDPopathies” encompass a large group of human disorders which include various cancers, neurodegenerative diseases, diabetes, and cardiovascular disease (Uversky, 2014). Frequently the misfolding of proteins including IDPs also leads to their aggregation, giving rise to the amyloidoses, in which more than 37 distinct proteins have been implicated (Chiti and Dobson, 2017).

MAP2 is an abundant dendritic MAP as well as an IDP which is closely related to Tau. It has been long appreciated for its roles in defining and maintaining dendritic structure; however, its neuropathological potential has been minimally explored. Evidence is beginning to suggest that MAP2 can be a similar pathogenic hub to other IDPs and MAPs, with various forms of MAP2 dysfunction having distinct causal factors and outcomes. For instance, ischemia leads to calpain-induced MAP2 cleavage which is thought to disrupt cytoskeletal integrity and may contribute to neuronal atrophy (Pettigrew et al., 1996). Similar MAP2 degradation occurs in prion disease (Guo et al., 2012). In contrast, in Huntington’s Disease, MAP2 splicing is altered, leading to an imbalance between high and low molecular weight MAP2 forms that is thought to contribute to the dendritic atrophy which characterizes the disorder (Cabrera and Lucas, 2017). Alternatively, our recent work in primary auditory cortex indicates that in schizophrenia, MAP2 is hyperphosphorylated, leading to changes in dendritic architecture (Grubisha et al., 2021).

The goal of this review is to summarize the current understanding of MAP2 function and regulation and review the existing evidence for MAP2 dysregulation in disorder, discussing potential forms and consequences of MAP2 pathology. From this, we contemplate a new conceptual framework for schizophrenia and other disorders as “MAP2opathies” in parallel to the tauopathies, bearing implications for their study and treatment.

## MAP2 Structure and Function

### Domains and isoforms of MAP2

**Figure 1:**
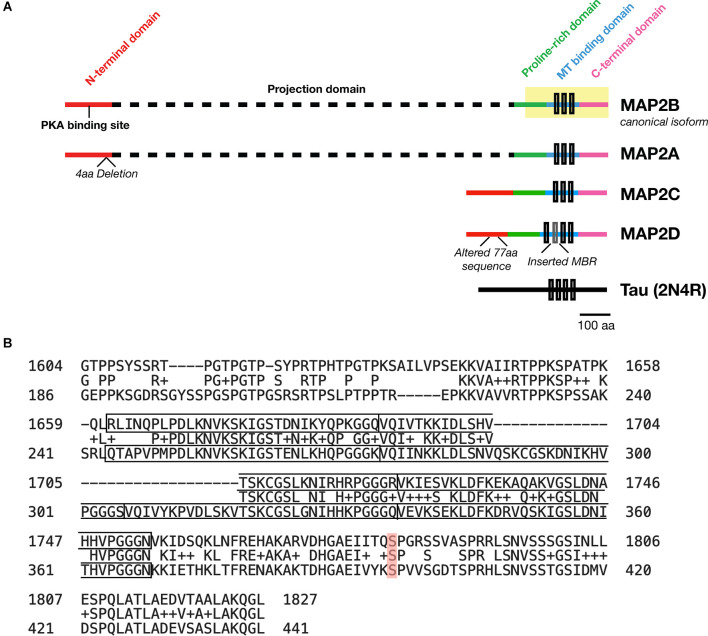
Domains of MAP2 and homology to MAP Tau. **(A)** Diagram depicting the domains of the four major isoforms of MAP2. Microtubule (MT)-binding repeats are denoted by black rectangles. Note that an additional MT binding repeat (MBR; gray box) is present in the low molecular weight (LMW) isoform MAP2D. Tau is also shown for size comparison. **(B)** Protein BLAST (https://blast.ncbi.nlm.nih.gov/Blast.cgi) alignment of the C-terminus (highlighted region of MAP2B in **A**) from MAP2B (Uniprot P11137-1; top) and 2N4R Tau (P10636-8; bottom). The MT-binding repeat sequences for each protein are indicated with solid boxes. The middle row indicates homologous or similar (+) residues. MAP2B S1782 (mentioned in the text) is highlighted in red. Figure generated in Microsoft Powerpoint.

MAP2 is comprised of five functionally distinct domains: the N-terminus, a projection domain, a proline-rich domain, the MT-binding domain, and the C-terminus (Figure 1A). The MT-binding domain in turn contains 3–4 MT binding imperfect repeats, though only the last of these seems to be necessary for the MT bundling activity of the protein (Ludin et al., 1996). Though this domain was originally identified as the critical domain for MT-binding functionality based on its affinity for bovine MTs (Lewis et al., 1988), subsequent data has suggested that it alone is not sufficient for full MT-binding of MAP2. Indeed, addition of flanking sequences from both the proline-rich and C-terminal domains is necessary to match full-length MT-binding activity in transfected HeLa cells (Ferralli et al., 1994). Though the precise mechanism of MAP2/MT interaction remains unknown, the “jaws” model proposed for MT binding by Tau (Gustke et al., 1994)—with which MAP2 shares 90% sequence homology (Figure 1B) —may provide a framework to visualize this interaction, wherein flanking domains are responsible for positioning MAP2/Tau on the MT while the MT-binding domain mediates tubulin polymerization.

In addition to facilitating MT binding, these flanking domains are likely to serve roles in mediating interactions with key binding partners. The closely homologous proline-rich domain of Tau, for instance, mediates interaction with SH3 domain-containing proteins, and MAP2 is known to interact with several such proteins such as the +TIP protein EB3 (Kapitein et al., 2011), Src and Grb2 (Lim and Halpain, 2000). The N-terminus of MAP2 also notably contains a binding site for the PKA regulatory subunit subtype II. As such, MAP2 is the primary dendritic A-kinase anchoring protein (AKAP; Zhong et al., 2009). In contrast, the projection domain appears to regulate MT spacing (Chen et al., 1992).

MAP2 has four major isoforms *in vivo* derived from alternative splicing: high molecular weight (HMW) MAP2A and MAP2B (the canonical isoform), and low molecular weight (LMW) MAP2C and MAP2D. These four MAP2 isoforms share four of the five functional domains, with the projection domain being selective to the HMW forms. In human, MAP2A possesses a 4-residue deletion relative to MAP2B, and MAP2D contains an additional MT-binding repeat and a modified 77-residue segment in the N-terminal domain relative to the other three isoforms (see Figure 1A). Whereas the subcellular localization of Tau is predominantly axonal (although some Tau has been observed in dendrites and dendritic spines), MAP2 are predominantly somatodendritic (Caceres et al., 1984), although MAP2C can be detected in axons (Meichsner et al., 1993), and MAP2D may be expressed in some glia (Doll et al., 1993). Additional splice variants—including protein coding transcripts—of MAP2 may also exist but remain uncharacterized (Howe et al., 2021).

Following transcription, *MAP2* mRNA localizes to neuronal dendrites for local translation (Garner et al., 1988). In rat neurons, translocation of this mRNA is dependent upon the *trans-*acting protein MARTA2—which has high affinity for a *cis*-acting 3’ UTR dendritic targeting element (Blichenberg et al., 1999)—as well as the kinesin KIF5 (Rehbein et al., 2000; Zivraj et al., 2013). Evidence suggests that local MAP2 protein translation in dendrites can be activity-dependent; prolonged high-frequency stimulation of the middle molecular layer of rat dentate gyrus causes an increase in MAP2 immunoreactivity in surrounding lamina, which is diminished by inhibition of protein synthesis (Steward and Halpain, 1999). This may be mediated by mTOR signaling, as rapamycin blocks tetanus-induced dendritic synthesis of MAP2 protein in hippocampal slice (Gong et al., 2006).

An enhanced selectivity of the HMW forms for the dendritic compartment may be due to presence of the projection domain, which inhibits MAP2A/B access to axons (Kanai and Hirokawa, 1995), perhaps *via* interactions with axonal initial segment proteins such as AnkyrinG (Hedstrom et al., 2008). Differential MT organization in dendrites and axons is subsequently thought to result from MAP2 vs. Tau binding (Chen et al., 1992). In addition to the dendritic shaft, MAP2 has also been observed in dendritic spines as early as 1989 by immunogold electron microscopy (Morales and Fifkova, 1989), though it is found less abundantly in spines in the absence of synaptic stimulus (see Section “Synaptic plasticity”).

MAP2 is expressed shortly after the switch from neuronal precursor to neuron, and its isoforms are differentially expressed across neurodevelopment. MAP2B is expressed throughout development; MAP2A levels increase during development; MAP2C levels decrease during development, although it remains present in adulthood (Jalava et al., 2007). As a result, HMW MAP2 forms the vast majority of MAP2 in adulthood. In addition, MAP2C is expressed in the mature testis where it is involved in spermatogenesis (Sun and Handel, 2011).

### Functions of MAP2

#### Interactions with cytoskeletal filaments

It has been long accepted that the primary function of MAP2, like Tau, is to bind MTs to increase their polymerization and bundling (Herzog and Weber, 1978; Stearns and Brown, 1979; Lewis et al., 1989). MAP2 is also commonly called an MT stabilizer, though importantly it does not suppress MT dynamics; rather, it reduces the frequency of depolymerizing “catastrophe” events in assembling MTs to enable their growth (Itoh and Hotani, 1994). This mechanism of MAP2 (and Tau; Baas and Qiang, 2019) can be thought of as a “dynamics-preserving” stabilization, as opposed to that of the “genuine” stabilizer MAP6, knockdown of which increases MT dynamics in the axonal compartment (Tortosa et al., 2017).

In addition to MTs, MAP2 can also bind and bundle actin, like Tau (Correas et al., 1990; Moraga et al., 1993; Roger et al., 2004). Thus, both proteins act as cross-linkers between the MT and actin networks (Mohan and John, 2015). Such crosstalk is important for various aspects of fundamental cellular functions such as cell motility and division. However, both proteins may affect actin filaments differently; for instance, phosphatidylinositol disrupts MAP2C- but not Tau-induced actin bundles (Yamauchi and Purich, 1993).

#### Process formation

The MT polymerizing and bundling effects of MAP2 facilitate process formation and maintain mature dendritic structure. Indeed, expression of MAP2 has repeatedly been shown to induce process formation in multiple heterologous cell lines (Edson et al., 1993; Leclerc et al., 1996). In neurons, MAP2 may act as the primary target of a number of endogenous agents which affect neurite structure *via* MT dynamics. For instance, pregnenolone—a neurosteroid with potential antidepressant and neuroprotective properties (Osuji et al., 2010; Marx et al., 2011, 2014)—enhances neurite growth by binding directly to MAP2 to simulate tubulin polymerization (Murakami et al., 2000; Fontaine-Lenoir et al., 2006). Melatonin increases neurite length, cellular MT content and MAP2 expression in a variety of contexts (Meléndez et al., 1996; Prieto-Gómez et al., 2008; Shu et al., 2016).

The neurite-forming and elongating effects of MAP2 can be disrupted by multiple interventions. The process appears to be negatively regulated by the projection domain, as MAP2B has a lower capacity for process formation than MAP2C in Sf9 cells (Bélanger et al., 2002). Expression of antisense oligonucleotides against MAP2 reduces the number of neurites and MTs within neurites in primary cortical cultures (Sharma et al., 1994). Similarly, *in vivo* knockout of MAP2 decreases dendritic length and MT density in hippocampal neurons (Harada et al., 2002). Expression of mutant MAP2 constructs can also be used to disrupt dendritic morphogenesis; overexpression of a phosphomimetic MAP2C construct which cannot bind to MTs (S319E/S350E/S382E; Ozer and Halpain, 2000) yields shortened neurites both in dissociated neuronal culture (Huang et al., 2013) and in Neuro-2a cells (Dehmelt et al., 2003) relative to wild-type MAP2C expression. Therefore, changes in MAP2 expression, splicing, or phosphorylation could drive pathological changes in dendritic morphology, namely in reduced dendritic outgrowth.

#### Synaptic plasticity

MAP2 disruption can also affect structural and functional plasticity of synapses. As shown in the work of Kim et al. (2020); HMW MAP2 is recruited to spines following chemical long-term potentiation (LTP) induction, whereas knockdown of HMW MAP2 impairs LTP induction, LTP-induced growth of dendritic spines, and AMPAR recruitment to spines. This function appears to be selective to the HMW forms of MAP2, as MAP2C-GFP fails to translocate into spines in an activity-dependent manner. Notably, knockdown of HMW MAP2 did not alter spine density in this study; however, we have recently established that at least one MAP2 phosphomimetic mutant (S1782E) is associated with a reduction in spine density in cortical pyramidal neurons of CRISPR knock-in mice (Grubisha et al., 2021). Thus, MAP2 can also contribute to spine formation and/or maintenance, however these functions may be compensated for by related cytoskeletal proteins (such as MAP1B; Teng et al., 2001) in the case of knockdown/knockout.

The role of MT dynamics in mediating the structural and functional plasticity of dendritic spines has become well-appreciated following the discovery that MTs transiently invade spines in an activity-dependent manner (Gu et al., 2008; Hu et al., 2008; Mitsuyama et al., 2008; Jaworski et al., 2009). Such invasions facilitate NMDAR-dependent spine head growth (Merriam et al., 2011), delivering cargo to the spine head in response to synaptic activity (McVicker et al., 2016). The link between regulation of MT dynamics and spine structure is further supported by findings that inhibition of MT polymerization leads to impaired LTP, regression of mature-appearing spines to immature spine shapes, and loss of spines (Jaworski et al., 2009). Thus, it is plausible that MAP2 mediates its effects on synaptic plasticity *via* MT dynamics. Indeed, the binding of MAP2 to MTs is itself an activity-dependent process (Vaillant et al., 2002), likely guided *via* phosphorylation (see below). Recent studies have also illuminated the importance of MT-actin crosstalk to spine morphogenesis, with actin remodeling at activated spines promoting MT entry (Merriam et al., 2013; Schätzle et al., 2018), although to date it is unknown if the MT-actin crosslinking function of MAP2 plays a role in this process.

In addition to its direct interaction with cytoskeletal networks, MAP2 also may mediate its effects on plasticity *via* its interactome. MAP2 creates local dendritic reservoirs of proteins critical to LTP/LTD, including PKA, EB3, RNA particles and polyribosomes (Lim and Halpain, 2000; Nielsen et al., 2002; Ostroff et al., 2002; Angenstein et al., 2005; Farah et al., 2005; Zhong et al., 2009; Kapitein et al., 2011; Sontag et al., 2012; Chirillo et al., 2019; Kim et al., 2020). MAP2 is known to mediate the relocalization of EB3 to the dendritic shaft following chemical long-term depression (cLTD) stimulus, concurrent with a reduction in MT invasion events (Kapitein et al., 2011). Conversely, translocation of HMW MAP2 to spine heads following chemical LTP (Kim et al., 2020) raises the possibility that MAP2 may contribute to activity-dependent protein trafficking into spines. Additionally, as an AKAP, MAP2 is thought to contribute to a spatial gradient of type II PKA formed between the dendritic shaft and spines following cAMP elevation, which in turn impacts synaptic strength and LTP induction (Zhong et al., 2009). Moreover, our own proteomic screen of the MAP2 interactome identified MAP2 interactions with RNA binding proteins regulating translation which served to inhibit protein synthesis (Grubisha et al., 2021), potentially impacting dendritic spine plasticity. Such proteins included FMR1, PCBP1–3, and hnRNPK, which has been previously shown to regulate spine density and LTP as well as mediate the effects of BDNF on dendritic mRNA metabolism and NMDAR function (Folci et al., 2014; Leal et al., 2017). MAP2 protein has previously been shown to interact directly with IMP1 *via* its KH domains, which facilitate nucleic acid binding in a number of RNA-binding proteins (Nielsen et al., 2002). Thus, at least some of the observed interactions with RNA-binding proteins may be direct as opposed to indirect associations *via* tubulin or associated motor proteins. However, these interactions remain to be verified.

#### Regulation of protein folding and transport

The association of MAP2 with ribosomal proteins is not a novel discovery (Farah et al., 2005) but has remained a relatively underappreciated aspect of the protein. We have found that this association has functional consequence, as overexpressed MAP2C inhibits protein synthesis in HEK cells (Grubisha et al., 2021). The mechanism of such regulation is unknown, but may depend on its association with RNA-binding proteins (see above). Overexpression of MAP2 may ultimately sequester such proteins to disrupt typical RNA trafficking and subsequently, protein synthesis. In addition to regulating protein synthesis, MAP2 has also been shown to exhibit chaperone-like qualities, preventing protein aggregation and facilitating enzyme refolding (Sarkar et al., 2004; Mitra et al., 2015). Of particular interest is the association MAP2 shares with Tau; MAP2 prevents the arachidonic acid-induced aggregation of Tau protein (Mitra et al., 2015), although the mechanism thereof is still unclear. MAP2 also regulates protein transport, predominantly by steric inhibition of the MT motors kinesin and dynein (Lopez and Sheetz, 1993; Hagiwara et al., 1994; Seitz et al., 2002). This can also contribute to selective axonal transport; using a sensory neuron model, Gumy et al. (2017) demonstrated that MAP2B generates a pre-axonal filtering zone controlling the activity of kinesin-1 and -5. Thus, MAP2 can shape the expression, conformation and transport of other proteins. However, these capacities remain understudied, particularly in the context of synaptic plasticity, where it may facilitate protein translocation into spines (Kim et al., 2020).

#### Neuronal death—marker or effector?

Neuron loss, regardless of cause, will be accompanied by corresponding loss of MAP2. However, in the context of induced brain injury, loss of MAP2 has been observed at early time points which precede neuronal death (Deshpande et al., 1992), raising the question of whether MAP2 loss may be involved casually in such death, presumably *via* disruption of the cytoskeletal network. MAP2 expression is significantly and consistently reduced early after induction of ischemia in both rodents (Dawson and Hallenbeck, 1996; Mages et al., 2021) and humans (Kühn et al., 2005), a loss which is thought to occur through calpain-mediated proteolysis (Pettigrew et al., 1996). However, the loss is also transient (Huh et al., 2003), suggesting that it does not correspond strictly to neuronal death. Moreover, MAP2 loss can be observed in regions of minimal cell death in mild traumatic brain injury models (Folkerts et al., 1998). Thus, whether MAP2 loss can precipitate or exacerbate neuronal death remains an open question which warrants further investigation, e.g., through characterization of neuronal survival following MAP2 knockout/knockdown, and/or rescue experiments exogenously expressing MAP2 during or post-injury. Such studies can illuminate whether MAP2 itself may represent a viable target for novel neuroprotective agents and strategies.

## Regulation of MAP2 Functions

Significantly, many MAP2 functions—such as its interactions with MTs and actin, as well as its chaperone-like properties—are regulated by phosphorylation. Phosphosites span the entirety of MAP2; however, phosphosites in the proline-rich, MT-binding and C-terminal domains share a higher degree of conservation across species and with Tau (Sánchez et al., 2000). Generally, phosphorylation tends to reduce the MT-binding affinity of MAP2 and therefore inhibit its MT bundling and polymerization-promoting functions, though the exact effects are site-specific. MAP2 phosphorylation is developmentally regulated (Riederer et al., 1995; Quinlan and Halpain, 1996; Sánchez et al., 2000), presumably mediating different phases of MT growth state and organization as neurons mature. Moreover, long-standing findings have established that MAP2 is also phosphorylated in response to synaptic activity that induces plasticity (Quinlan and Halpain, 1996; Li et al., 2016). This activity-dependent phosphorylation appears to be mediated at least in part by the Ras-MAPK pathway (Llansola et al., 2001; Kim et al., 2020), though to our knowledge, activity-dependent phosphosites of MAP2 have not yet been confirmed. Conversely, sensory deprivation appears to affect MAP2 phosphorylation as well; for instance, olfactory restriction sharply reduces immunoreactivity of AP18, a MAP2 antibody which recognizes pS136 (Philpot et al., 1997).

Other kinases known to phosphorylate MAP2 include PKA, CaMKII, PKC, GSK-3β, CDKs, and MARKs (Sánchez et al., 2000) as well as JNK1. JNK1 represents a notable exception to the general principle of MAP2 phosphorylation in that its phosphorylation of several proline-rich domain residues (T1619, T1622, and T1625) increases—rather than decreases—the protein’s binding to MTs, as well as enhances dendritic arborization (Komulainen et al., 2014). This diversity of upstream regulators allows highly precise regulation of MAP2 function under a variety of cellular conditions. Notably, while phosphosites in MAP2 are frequently conserved in Tau, the position of sites phosphorylated in each MAP by a single kinase can differ, even in regions of high homology. This is the case for PKA, which mainly phosphorylates MAP2C at S435, whereas its major phosphosite in Tau is S214 (Jansen et al., 2017). Thus, the two can be differentially regulated by shared upstream kinases.

Interestingly, upstream kinases of MAP2 overlap substantially with those implicated in neuropsychiatric and neurodegenerative disorders. For instance, MAPK3 (ERK1), a component of the Ras-MAPK pathway, has been identified as the probable causal gene underlying the association signal at its locus in the largest schizophrenia genome-wide association study conducted to date (Schizophrenia Working Group of the Psychiatric Genomics Consortium et al., 2020). MAPK3 is also present at the 16p.11.2 locus, deletion of which is associated with autism (Pucilowska et al., 2015). GSK-3β, another kinase of MAP2, is a major regulator of neuronal development and has been strongly implicated in Alzheimer’s disease (Maqbool et al., 2016). It is also thought to be a major effective target of various antipsychotics, antidepressants and mood stabilizers such as lithium (Beaulieu et al., 2009). PKA, a binding partner and kinase of MAP2, acts downstream of voltage-gated calcium channels (Davare et al., 1999), which are now clearly identified by unbiased genomic studies as contributing to risk in schizophrenia, bipolar disorder, depression, and autism spectrum disorders (Psychiatric GWAS Consortium Bipolar Disorder Working Group, 2011; Lu et al., 2012; Schizophrenia Working Group of the Psychiatric Genomics Consortium, 2014; Rao et al., 2016).

In addition to phosphorylation, MAP2 is likely regulated through other PTMs. Glycosylation (Ding and Vandré, 1996), ADP-ribosylation (Scaife et al., 1992), and lysine acetylation (Hwang et al., 2016) of MAP2 have all been described, though their functions are unclear. One study found that phosphorylation of MAP2 by GSK3β and/or PKA inhibited its O-glycosylation by 50%-90% (Khatra et al., 2013), suggesting that these regulatory mechanisms share an inverse relationship. Similarly, Tau is significantly less O-GlcNAc glycosylated when hyperphosphorylated (Lefebvre et al., 2003; Robertson et al., 2004). Beyond PTMs, functions of MAP2 are also regulated by alterative splicing. Besides the differences between HMW and LMW MAP2 described here (see Section “Domains and isoforms of MAP2”), alternative splice variants (Howe et al., 2021) have yet to be confirmed and/or characterized, but could also have distinct functions. Finally, interacting partners, including proteins and other biomolecules, can affect the actions of MAP2. For instance, as previously mentioned, Tau and phosphatidylinositol influence the MT-binding and actin-bundling properties of MAP2, respectively. Phosphatidylinositol additionally inhibits its MT assembling ability (Yamauchi and Purich, 1987).

## MAP2 in Disorder

The first half of this review has highlighted the diverse roles MAP2 plays in the development and maintenance of neuronal function, including in neurite extension/outgrowth, synaptic plasticity, and protein folding/transport. We have also described how these functions can be modified by a series of mechanisms, including downregulation/knockdown, splicing variants, and PTMs. Below we review the evidence of dysregulated MAP2 present in neuropsychiatric and neurodegenerative disorders (summarized in Table 1), and further discuss how MAP2 may contribute to their characteristic endophenotypes based on the known functions of the protein.

**Table 1:** Evidence of MAP2 dysfunction in neuropsychiatric and neurodegenerative disorder.

**Disorder**	**Region**	**Finding**	**References**
Schizophrenia	DLPFC	Reduced MAP2-IR intensity	DeGiosio et al. (2019)
	Lateral intraparietal cortex	Reduced MAP2-IR intensity	DeGiosio et al. (2019)
	Primary visual cortex	Reduced MAP2-IR intensity	DeGiosio et al. (2019)
	Hippocampus	Qualitative absence of MAP2-IR in subset of subjects	Arnold et al. (1991) and Rosoklija et al. (2005)
	Olfactory bulb	Reduced MAP2-IR intensity	Rioux et al. (2004)
	DLPFC	Reduced MAP2-IR+ area fraction	Somenarain and Jones (2010)
	Anterior cingulate cortex (BA32)	Reduced MAP2-IR+ area fraction	Jones et al. (2002)
	Primary auditory cortex	Reduced MAP2-IR intensity	Shelton et al. (2015) and McKinney et al. (2019)
	Primary auditory cortex	Increased MAP2 phosphorylation	Grubisha et al. (2021)
	Hippocampus	Reduced MAP2-IR by phospho-specific antibody	Cotter et al. (1997)
	Corpus callosum	Increased MAP2 phosphorylation	Saia-Cereda et al. (2016)
Autism spectrum disorders	DLPFC	Reduced MAP2-IR+ neurons/dendrites	Mukaetova-Ladinska et al. (2004)
	Neocortex	Qualitative reduction of MAP2-IR	Kaufmann et al. (1995)
	n/a	2q34 deletion encompasses *MAP2* gene and causes Rett-like features	Pescucci et al. (2003) and Westphal et al. (2018)
Mood disorders major depressive/bipolar disorders	Subiculum	Qualitative absence of MAP2-IR in subset of subjects	Rosoklija et al. (2005)
	DLPFC	Qualitative reduction of MAP2-IR	Kang et al. (2012)
	DLPFC	Reduced MAP2 protein	Kang et al. (2012)
	DLPFC	Decreased MAP2 phosphorylation at S233, S1031	Martins-de-Souza et al. (2012)
	Various regions	Reduced MAP2 mRNA in males with major depressive disorder	Labonté et al. (2017)
	Anterior cingulate cortex	Reduced MAP2 protein	Föcking et al. (2016)
Huntington’s Disease	Striatum	Altered MAP2 splicing	Cabrera and Lucas (2017)
	Striatum	Qualitative loss of dendritic MAP2-IR	Cabrera and Lucas (2017)
	DLPFC	Reduced MAP2-IR+ area fraction	Somenarain and Jones (2010)
Prion Disease	n/a brain homogenate	Reduced MAP2 protein (coincident with increase in calpain)	Guo et al. (2012)
Tauopathy	n/a	MAP2 phosphopeptides (T350, S1702, S1706) identified in purified NFTs	Rudrabhatla et al. (2011)

Evidence of MAP2 dysfunction in these disorders includes various irregularities in MAP2 immunoreactivity, expression level, and/or modification.

### MAP2 in neuropsychiatric disorder

#### Schizophrenia

Schizophrenia is a psychiatric disorder characterized by psychotic symptoms as well as a variety of cognitive dysfunctions. Reduced MAP2 immunoreactivity (MAP2-IR) has been reported in diverse brain regions in schizophrenia and has been described as a “molecular hallmark” of the disorder (Marchisella et al., 2016). This observation has been made in the subiculum, the olfactory bulbs, entorhinal cortex, BA9, BA32 and, in our studies, dorsolateral prefrontal cortex (DLPFC), lateral intraparietal cortex, primary visual cortex, and primary auditory cortex (BA41; Arnold et al., 1991; Jones et al., 2002; Rioux et al., 2004; Rosoklija et al., 2005; Somenarain and Jones, 2010; Shelton et al., 2015; DeGiosio et al., 2019; McKinney et al., 2019). The reduced MAP2-IR does not appear to result from reductions in neuron number/density, as this remains unchanged in areas where MAP2-IR loss is profound (Jones et al., 2002; Dorph-Petersen et al., 2009; Somenarain and Jones, 2010). In our own studies we have also excluded potential effects of several clinical and technical confounds such as postmortem interval (the time from death until tissue fixation), duration of tissue freezer storage, use of psychotropic medications, or substance use status at time of death (Shelton et al., 2015; DeGiosio et al., 2019; McKinney et al., 2019). While lower tissue pH (a possible indicator of brain health prior to death) was correlated with lower MAP2-IR in one study, this effect did not account for reductions present across the cortex in schizophrenia (DeGiosio et al., 2019). As previously alluded to (see Section “Neuronal death—marker or effector?”), cerebral ischemia/hypoxia have previously been shown to reduce numbers of MAP2-IR-positive neurons in human neocortex and hippocampus; however, although causes of death associated with cerebral hypoxia or ischemia were more common in our schizophrenia subjects, they also did not explain the differences in MAP2-IR (DeGiosio et al., 2019). These analyses indicate that MAP2-IR deficit can represent a selective impairment in MAP2 that is not simply consequential to common factors affecting neuronal health.

With the additional power available from a combined cohort of 45 schizophrenia subjects paired with non-psychiatric comparison subjects, we were able to conduct formal mediation tests demonstrating that lower MAP2-IR mediated reductions in the density of dendritic spines in primary auditory cortex, suggesting that pathology of MAP2 is related, potentially in a causal manner, to pathogenic processes. Interestingly, in this study we determined that MAP2 protein levels were not reduced (McKinney et al., 2019). Moreover, MAP2 mRNA levels are unchanged in the hippocampal formation in schizophrenia subjects (Law et al., 2004). Therefore, MAP2-IR loss in schizophrenia does not appear to reflect reductions in mRNA/protein levels. Instead, MAP2 may undergo aberrant PTM to change its function. This could also preclude homeostatic compensation by other MAPs, previously proposed to occur in MAP2 knockout mice, which have shown that MAP2 expression is dispensable to mouse survival (Teng et al., 2001; Harada et al., 2002).

In support of this idea, several studies have suggested that the phosphorylation state of MAP2 is altered in schizophrenia. The immunohistochemical studies of Cotter et al. (1997) showed a trend toward decreased immunoreactivity by Ab305—a MAP2 antibody which recognizes pT1616/pT1619 in the proline-rich domain—in hippocampus of individuals with schizophrenia. The findings of Saia-Cereda et al. (2016) indicate that MAP2 phosphorylation is globally altered in the corpus callosum of schizophrenia subjects. In addition, using phosphoproteomics methods we have recently shown that MAP2 is differentially phosphorylated in primary auditory cortex, with phosphorylation events tending to be upregulated in disorder. Moreover, in this study we demonstrated that a subset of identified MAP2 phosphopeptides were significantly correlated with dendritic spine density and synaptic protein levels in BA41 (Grubisha et al., 2021).

This provided the first evidence indicating a potential pathogenic role for MAP2 phosphorylation in schizophrenia. As a proof of concept, we showed that a MAP2 mutant mimicking the most highly upregulated phosphorylation event identified, pS1782 (pS426 in MAP2C; Figure 1B), reduced the MT binding affinity of the protein, and that CRISPR mice harboring this mutation displayed reduced spine density and dendritic complexity in auditory cortex (Grubisha et al., 2021), paralleling findings from postmortem tissue (reviewed in Glausier and Lewis, 2013). This indicates that at least one schizophrenia-associated MAP2 phosphorylation event may be capable of causing dendritic pathology *in vivo*. Interestingly, this residue is homologous to S396 in Tau- a residue which is associated with AD and regulates Tau localization as well as LTD (Bramblett et al., 1993; Mondragón-Rodríguez et al., 2014; Regan et al., 2015; Xia et al., 2015; Wesseling et al., 2020). We further found that levels of the pS1782-bearing phosphopeptide were significantly lower in schizophrenia subjects on antipsychotic medication at time of death, suggesting that this phosphorylation event may represent a target of antipsychotic treatment, although the effect was not recapitulated in a monkey model of long-term antipsychotic exposure (Grubisha et al., 2021). In summary, aberrant MAP2 phosphorylation in schizophrenia may both underlie the profound reductions in MAP2-IR observed postmortem and have direct consequences for neuronal structure and function.

#### Autism

Autism spectrum disorders are neurodevelopmental disorders characterized by alterations in social communication and repetitive behaviors, as well as varying levels of intellectual disability. In several case reports of intellectual disability and autism, reduced numbers of MAP2-IR+ neurons and dendrites has been noted, without a change in total neuron number (Kaufmann et al., 1995; Mukaetova-Ladinska et al., 2004). Interestingly, a significant change in MAP2 expression was noted in a recent proteomic analysis of autism (though this did not survive correction for false discovery rate; Abraham et al., 2019). Moreover, case study reports of rare 2q34 deletions—which encompass the *MAP2* gene—have described autism-like outcomes (Pescucci et al., 2003; Westphal et al., 2018). Though inconclusive, these data raise the possibility that MAP2 levels are reduced in at least some cases of autism. Such change, however, is likely to originate post-transcriptionally, as mRNA levels appear to be unaltered (Gandal et al., 2018).

A depletion of MAP2, even partial, could feasibly contribute to endophenotypes which characterize autism spectrum disorders, particularly with respect to synaptic plasticity. As described earlier, knockdown of MAP2 demonstrably impairs functional and structural plasticity of dendritic spines (Kim et al., 2020), which can be expected to alter learning and memory. Indeed, deficits in verbal learning/memory and working memory are among the most consistently impaired cognitive domains in adults with autism (Velikonja et al., 2019). Additionally, in postmortem brain tissue from individuals with autism, dendritic spine morphology shifts towards more immature phenotypes (Martínez-Cerdeño, 2017), which could result from a lack of MAP2-mediated activity-dependent spine growth. This may warrant further investigation of MAP2 abundance and function in autism.

#### Mood disorders

MAP2-IR reduction has additionally been reported in the DLPFC of individuals with major depressive disorder (Kang et al., 2012) and in the hippocampus of major depression and bipolar disorder subjects (Rosoklija et al., 2005). The origin of this reduction is unclear. In the work of Kang et al. (2012), reduced DLPFC MAP2 levels were observed by western blot; however, it is unclear from these data if this is solely the result of reduced neuron density, which has also been noted in the region (Rajkowska et al., 1999). In contrast, microarray data indicates no change in MAP2 mRNA expression in depression (Seney et al., 2018). However, a separate study utilizing RNA sequencing to study sex-specific transcriptomic changes in depression identified a male-specific reduction in MAP2 mRNA level in multiple cortical regions (Labonté et al., 2017). Decreased MAP2 was also recently observed in bipolar disorder *via* proteomic analysis of the postsynaptic density (Föcking et al., 2016)—though not, to date, at the whole homogenate level—while it fails to exhibit change at the mRNA level by RNA sequencing (Gandal et al., 2018).

Two MAP2 phosphorylation events in the projection domain (pS233 and pS1031) have been reported to be decreased (case:control ratio = 0.82–0.83) in DLPFC of individuals with major depression, though these changes did not remain significant after controlling for false discovery rate (Martins-de-Souza et al., 2012). Interestingly, however, chronic antidepressant treatment has been observed to affect MAP2 expression and/or phosphorylation and subsequently, tubulin polymerization kinetics. Subchronic treatment of naïve rats with imipramine increases MAP2-IR in hippocampus (Iwata et al., 2006), while treatments with fluvoxamine, desipramine, maprotiline, and citalopram increase MAP2 phosphorylation (as assessed using phosphospecific antibodies or ^32^P incorporation), in turn suppressing tubulin polymerization (Miyamoto et al., 1995, 1997; Perez et al., 1995). It is unknown if and how such modification of MAP2 might contribute to antidepressant effects; however, previous data indicates that transient phosphorylation of MAP2 could support the dendritic outgrowth observed in response to antidepressant treatment (Seo et al., 2014). Indeed, ^32^P incorporation in MAP2 has previously been shown to correlate with dendritic arborization across time in cultured hippocampal neurons (Díez-Guerra and Avila, 1993). This observation was soon after substantiated by data indicating that treatment with protein kinase inhibitors reduces dendritic branching in these cells, while protein phosphatase inhibitors increase branching (Audesirk et al., 1997). However, our work on MAP2 S1782E—in which pseudo-phosphorylation of MAP2 reduced dendritic length and branching (Grubisha et al., 2021)—suggests that effects of MAP2 phosphorylation on dendritic arborization are complex and could depend on site, brain area and/or duration of signal. These data warrant further investigation of MAP2 phosphorylation state in depression, and of the specific interactions between MAP2 and antidepressants to better understand their mechanism of action.

### MAP2 in neurodegenerative disorder

#### Huntington’s disease

Huntington’s disease is a heritable neurodegenerative disorder caused by mutation in huntingtin, a microtubule-associated protein involved in axonal transport. The work of Cabrera and Lucas (2017) established that MAP2 splicing is altered in striatum of Huntington’s subjects, favoring the LMW forms and reducing overall MAP2 levels. The authors proposed that this aberrant splicing of MAP2 results from altered activity of splicing factor SRSF6, previously implicated in the disease. The loss of MAP2 protein was accompanied by a substantial loss of MAP2-IR in the region, complimenting prior work showing reduced MAP2-IR in Brodmann area 9 of Huntington’s subjects (Somenarain and Jones, 2010).

As described above (see Section “Domains and isoforms of MAP2”), the HMW and LMW forms of the protein differ by inclusion or exclusion of the projection domain. This yields differential subcellular localization patterns and MT spacing characteristics. Additionally, they appear to differ in their ability to support activity-dependent spine plasticity, with HMW forms selectively translocating into spines following chemical LTP stimulus (Kim et al., 2020). In Huntington’s, such MAP2-mediated plasticity may be diminished, with consequences for learning and memory. Additionally, LMW forms appear to have greater affinity for neurite formation than HMW forms (Bélanger et al., 2002). Thus, the shift towards LMW MAP2 could be expected to yield more dendrites, and indeed this has been reported in prefrontal cortex of a small cohort of Huntington’s subjects (Sotrel et al., 1993). Therefore, aberrant MAP2 splicing may represent a crucial precipitant to the structural and functional abnormalities seen in neurons affected by Huntington’s disease.

#### Prion disease

Prion diseases are a group of transmissible neurodegenerative diseases caused by prions—misfolded proteins which can transmit their misfolded shape onto normal variants of the same protein, causing aggregation and altered protein function. Work by Guo et al. (2012) described significant reductions of MAP2 protein in scrapies-infected hamsters, attributable to a concomitant increase in the protease calpain. Interestingly, this parallels findings from brain injury models, such as ischemia and traumatic spinal cord injury, which also describe calpain-mediated MAP2 degradation (Pettigrew et al., 1996; Springer et al., 1997). Aβ oligomers can similarly induce such degradation (Fifre et al., 2006). Further, one recent study has demonstrated the downregulation of MAP2 protein in SMN-deficient motor neuron-like cells modeling spinal muscular atrophy, which the authors speculate may also be due to calpain hyperactivity (Özer et al., 2022). Thus, MAP2 appears to be a frequent target of calpain to mediate MT destabilization and potentially cell death under a variety of circumstances. Further study can clarify what therapeutic value MAP2 may have in preventing neuron loss (see Section “Neuronal death—marker or effector?”).

#### Tauopathies

The tauopathies are a group of neurodegenerative disorders characterized by the hyperphosphorylation of MAP Tau and its subsequent assembly into neurofibrillary tangles (NFTs). MAP2 has generally not been considered as a potential pathogenic agent in the tauopathies, as its presence in NFTs has been a subject of some debate (Xie et al., 2014) and it forms no analogous aggregates. However, recent works have called into question whether NFTs themselves drive neuropathology, or if they are by products of pathology which is driven by soluble tau forms (Kopeikina et al., 2012). Indeed, the work of Xie et al. (2014) showed that while MAP2 and Tau have different aggregation properties, pan-neuronal expression of either can elicit severe neurotoxic effects. In this revised framework, interactions between MAP2 and soluble Tau may be important in shaping tauopathy. MAP2 and Tau have been known to cross-regulate each other; for instance, hyperphosphorylated Tau can sequester HMW MAP2 and thereby inhibit MT assembly (Alonso et al., 1997). Conversely, MAP2 prevents the arachidonic acid-induced aggregation of Tau, though MAP2 phosphorylation impairs this chaperoning ability (Mitra et al., 2015). Thus, MAP2 and Tau seem to share a reciprocal relationship whereby dysregulation of one can lead to that of the other. Mass spectrometry methods have revealed the presence of MAP2 phosphopeptides in purified Alzheimer’s NFTs (Rudrabhatla et al., 2011), indicating that MAP2 may be hyperphosphorylated in the disorder. Thus, MAP2 remains an active element to consider in the pathogenesis of tauopathies. Better understanding the origin of its aberrant phosphorylation in tauopathies and the nature of its interactions with Tau could lead to new therapeutic targets for the prevention of Tau aggregation.

## The “MAP2opathy”—Limitations and Future Directions

MAP2 is often viewed solely as an endpoint marker of neuronal health. However, it is also a critical cytoskeletal regulator, dysfunction of which drastically affects neuronal structure and function. We have here described the known roles of MAP2 in neurite outgrowth, synaptic plasticity and protein folding/transport, as well as mechanisms of its regulation, including most prominently alternative splicing and phosphorylation. As such, MAP2 is poised to become dysregulated in various ways, potentially leading to distinct, pleiotropic effects. We have reviewed the current evidence for alterations in MAP2 function present in various neuropsychiatric and neurodegenerative disorders such as schizophrenia and Huntington’s disease. MAP2 pathology appears to originate at different levels across these disorders; for instance, Huntington’s is marked by aberrant RNA splicing, autism may be associated with lowered MAP2 protein abundance, while schizophrenia is distinguished by aberrant post-translational modification of MAP2.

**Figure 2:**
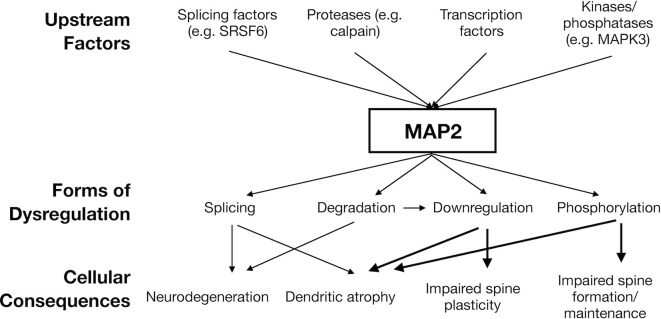
*Hypothesized model of “MAP2opathy”.* Genetically- or environmentally-precipitated upstream risk factors are expected to affect MAP2 in a variety of ways, leading to diverse, overlapping cellular consequences. Thick arrows indicate causal relationships established in *in vivo* models (Harada et al., 2002; Kim et al., 2020; Grubisha et al., 2021). Thin arrows indicate noted correlational relationships (Pettigrew et al., 1996; Guo et al., 2012; Cabrera and Lucas, 2017). Figure generated in Microsoft Powerpoint.

This parallels the multiple distinct mechanisms by which tau can become dysregulated in tauopathy, which is not limited to mutations in MAPT genes, but also includes protein modification (particularly phosphorylation), *cis/trans* isomerization, and more. Thus, a “MAP2opathy” may similarly exist, characterizing varied neurodevelopmental and neurodegenerative disorders; akin to Tau, MAP2 could represent a molecular bottleneck situated downstream of genetic and environmental risk factors and upstream of neuronal pathology (Figure 2). This would position MAP2 as a flexible therapeutic target, able to ameliorate pathology while remaining effective in a large proportion of affected individuals regardless of their unique set of risk factors. However, classification of MAP2opathy as a genuine proteinopathy is still premature and awaits further research. Table 2 outlines various forms of evidence in favor of classification of a proteinopathy, comparing the existing evidence in the case of either tau or MAP2. An overall lack of genetic evidence implicating MAP2 mutation in disorder distinguishes it from tau and requires consideration. This could be due to the role of LMW MAP2 in spermatogenesis (Sun and Handel, 2011), which may make the gene mutation-intolerant [loss of function observed/expected upper bound fraction (LOEUF) = 0.1 (Karczewski et al., 2020)] and limit the ability of organisms to transmit MAP2 mutations that could later cause neuropathology. Instead, pathogenic MAP2 is more likely to arise from integrated upstream processes including kinase/phosphatase function, splicing machinery and proteolytic processes, analogous to those at play in tauopathy.

**Table 2:** Evidences of proteinopathy for tau vs. MAP2.

**Evidence of proteinopathy**	**Tau**	**MAP2**
Mutation of the encoding gene is associated with disease	Mutations in *MAPT* gene lead to FTDP-17 as well as other neurodegenerative disorders similar to Pick’s disease, corticobasal degeneration and supranuclear palsy (Strang et al., 2019)	Autism-associated rare 2q34 microdeletions encompass the *MAP2* gene (Pescucci et al., 2003); however, **mutations selective to *MAP2* have not to date been associated with disorder**
Modifications of transcript or protein are associated with disease	Tau protein hyperphosphorylation is a hallmark of tauopathy, contributing to its aggregation/formation of NFTs (Noble et al., 2013). Missplicing of Tau pre-mRNA- in some cases associated with *MAPT* mutation- is also associated with various tauopathies (Park et al., 2016)	Changes in expression, post-translational modification and/or splicing of MAP2 have been described in various disorders (see Table 1)
Experimental manipulation of the protein causes disease-associated pathology	Mutant Tau mouse lines such as rTg4150 can replicate key features of tauopathy including neuron loss and memory impairments (Denk and Wade-Martins, 2009). Phosphomimicry of Tau can lead to learning and memory deficits in *Drosophila* and mice (Beharry et al., 2013; Di et al., 2016)	MAP2 knockdown can lead to impairments in LTP and dendritic outgrowth, which are implicated in neurodevelopmental and neurodegenerative disorder (Kim et al., 2020). Mimicry of a schizophrenia-associated MAP2 phosphorylation event leads to reduced dendritic arborization and spine density (Grubisha et al., 2021). **Other disease-relevant modifications of MAP2, such as altered splicing or cleavage, have not been modeled. Behavioral/cognitive outcomes of mutant MAP2 animals have not yet been reported**
Protein dysregulation precedes functional impairments	Tau deposition precedes clinical symptoms of disorder in humans (Jack et al., 2010)	**Time course of MAP2 PTMs, splicing alterations, etc remain unknown**
Degree of protein dysregulation scales with disease pathology	Burden of tau PET binding correlates with cognitive symptoms and severity of regional atrophy (Cho et al., 2016). Higher NFT burden is also associated with faster cognitive decline (Jefferson-George et al., 2017)	MAP2 phosphopeptide levels correlate with dendritic spine density and socioeconomic status in individuals with schizophrenia (Grubisha et al., 2021). Additionally, MAP2-IR loss statistically mediates dendritic spine loss in primary auditory cortex of subjects with schizophrenia (McKinney et al., 2019). **Associations of MAP2 expression/phosphorylation with pathology in other disorders remains uninvestigated**

Bold face text highlights current gaps in knowledge regarding MAP2 pathology.

Existing data regarding MAP2 pathology is largely limited to correlational study, leaving open the possibility that changes to MAP2 immunoreactivity, expression or modification do not contribute to pathogenesis, but instead are merely incidental changes incurred by upstream processes. Despite clear evidence that disruptions to MAP2 expression and/or modification can lead to changes in dendritic and synaptic structure and function (Sharma et al., 1994; Ozer and Halpain, 2000; Harada et al., 2002; Akulinin and Dahlstrom, 2003; Dehmelt et al., 2003; Huang et al., 2013; Kim et al., 2020; Grubisha et al., 2021), this largely remains to be demonstrated in disease-relevant contexts. For example, our modeling of a schizophrenia-associated MAP2 phosphorylation event demonstrates a potential causal role of dysregulated MAP2 in the altered dendritic outgrowth which characterizes this disorder (Grubisha et al., 2021). Similarly, the altered splicing of MAP2 observed in Huntington’s might be modeled experimentally by manipulation of SRSF6 splicing factor, and rescue experiments can be performed by exogenous expression of HMW MAP2 to observe consequential effects on dendritic growth. Better understanding patterns of calpain-mediated cleavage of MAP2 could enable modeling of its degradation using truncation-inducing mutations or overexpression of truncated MAP2 constructs to explore roles of MAP2 degradation in neuronal atrophy of prion diseases or brain injury. MAP2 phosphorylation events observed in NFTs also have yet to be modeled, which could be used to illuminate what roles—if any—MAP2 phosphorylation plays in Tau aggregation/pathology.

Additionally, recent works revealing new or understudied functions of MAP2, such as that in LTP induction and AMPAR translocation (Kim et al., 2020), neuronal polarization *via* axonal filtering (Gumy et al., 2017), and our own work on phosphomimetic MAP2 (Grubisha et al., 2021) make clear that there is still much to learn about the basic biology of MAP2 protein. Firstly, thorough characterization of MAP2 PTM state on a residue-specific level in both healthy and diseased contexts will prove informative and can be readily achieved through modern mass spectrometry methods. Roles of such modifications in cytoskeletal filament organization, neurite outgrowth, synaptic plasticity and neuronal viability remain to be examined. Such studies will likely benefit from the extant literature regarding Tau, as these proteins structurally and functionally parallel one another—for example, through conserved phosphosites such as S396Tau/S1782MAP2 (Grubisha et al., 2021). Further, a bias exists in the literature favoring the *in vitro* study of immature MAP2c relative to mature, HMW forms of the protein, likely due to its ease of production and purification. However, these forms have differential structure and functions that remain to be fully understood. Such distinctions will provide insights to the developmental regulation of MAP2 functions. In conclusion, future studies of MAP2 will not only enhance our understanding of basic neurobiology but can also shed light on pathogenesis of a potential therapeutic target in neuropsychiatric and neurodegenerative disorders.

## Data Availability Statement

The original contributions presented in the study are included in the article, further inquiries can be directed to the corresponding author.

## Author Contributions

RD and RS conceived the idea for this manuscript. RD performed the literature search and drafted the work with assistance from RS. MG, MM, BM, and CC critically revised the work. All authors contributed to the article and approved the submitted version.

## Funding

This work was supported by National Institute of Mental Health (grant numbers: MH116046, MH071533, MH118497, and MH125235), National Institute on Aging (grant number: AG027224), and National Institutes of Health (grant number: GM97082).
